# Differential Epigenetic Effects of Atmospheric Cold Plasma on MCF-7 and MDA-MB-231 Breast Cancer Cells

**DOI:** 10.1371/journal.pone.0129931

**Published:** 2015-06-04

**Authors:** Sung-Bin Park, Byungtak Kim, Hansol Bae, Hyunkyung Lee, Seungyeon Lee, Eun H. Choi, Sun Jung Kim

**Affiliations:** 1 Department of Life Science, Dongguk University-Seoul, Goyang, Korea; 2 Plasma Bioscience Research Center, Kwangwoon University, Seoul, Korea; University of Salerno, Faculty of Medicine and Surgery, ITALY

## Abstract

Cold atmospheric plasma (plasma) has emerged as a novel tool for a cancer treatment option, having been successfully applied to a few types of cancer cells, as well as tissues. However, to date, no studies have been performed to examine the effect of plasma on epigenetic alterations, including CpG methylation. In this study, the effects of plasma on DNA methylation changes in breast cancer cells were examined by treating cultured MCF-7 and MDA-MB-231 cells, representing estrogen-positive and estrogen-negative cancer cells, respectively, with plasma. A pyrosequencing analysis of *Alu* indicated that a specific CpG site was induced to be hypomethylated from 23.4 to 20.3% (p < 0.05) by plasma treatment in the estrogen-negative MDA-MB-231 cells only. A genome-wide methylation analysis identified “cellular movement, connective tissue development and function, tissue development” and “cell-to-cell signaling and interaction, cell death and survival, cellular development” as the top networks. Of the two cell types, the MDA-MB-231 cells underwent a higher rate of apoptosis and a decreased proliferation rate upon plasma treatment. Taken together, these results indicate that plasma induces epigenetic and cellular changes in a cell type-specific manner, suggesting that a careful screening of target cells and tissues is necessary for the potential application of plasma as a cancer treatment option.

## Introduction

Non-thermal atmospheric pressure plasma is ionized media that contains a mixture of active particles, including electrons, ions, free radicals, reactive molecules and photons [1, 2]. Part of this mixture consists of reactive oxygen and nitrogen species, such as ozone, superoxide, hydroxyl radicals, singlet oxygen, atomic oxygen, nitric oxide, nitrogen dioxide, nitrite, and nitrates [3, 4].

Plasma has recently emerged in multiple medical applications, having been shown to be highly effective in wound healing and blood coagulation, as well as in the treatment of various diseases, including cancer [5, 6]. For example, in ovarian cancer, chronic chemo-resistant ovarian cancer cells in plasma-activated medium showed decreased cell viability. Furthermore, in a murine subcutaneous tumor-formation model, the injection of plasma-activated media resulted in an inhibition of the ovarian cancer cell-inoculated tumor [7]. In head and neck carcinomas, cold plasma selectively impaired cancer cell lines through non-apoptotic mechanisms, while having a minimal effect on normal oral cavity epithelial cell lines [8]. In breast cancer, it was demonstrated that plasma modified for specific conditions selectively ablated metastatic breast cancer cells *in vitro* by inhibiting the migration and invasion of the cells, while minimally influencing healthy bone marrow mesenchymal stem cells [1]. In addition, cold plasma has been proven to be effective in various other cancer types, including glioma [9], melanoma [10], and pancreatic cancer [11], inducing apoptosis and/or senescence of the cancer cells.

The molecular mechanisms underlying the changes in cellular activity by plasma have been explained in a few cases. For example, cold plasma-treated oral cavity squamous cell carcinoma was arrested at the sub-G1 phase and the arrest was associated with DNA damage and the ATM/p53 signaling pathway in SCC25 cells [12]. In *in vitro* and *in vivo* studies with a wound healing model, plasma induced expression of the key genes crucial for the wound healing response, like IL-6, IL-8, MCP-1, TGF-b1, and TGF-b2 [13]. Recently, it was found that plasma induced apoptosis of p53-mutated cancer cells by activating ROS (reactive oxygen species) stress-response pathways [14].

DNA methylation at CpG sites functions in the epigenetic regulation of gene activity whereby tumor suppressors, or oncogenes, are hyper- or hypomethylated during tumorigenesis [15, 16]. Thus far, different forms of radiation that are currently used for medical applications, such as x-rays, γ-rays, and proton beams, have been known to induce methylation changes in cancer-related genes [17–19]. Despite a large body of experimental evidence regarding gene expression, as well as cellular activity, few studies have been performed thus far to investigate the effect of plasma on epigenetic changes, such as DNA methylation, which is one of the main factors contributing to tumorigenesis. Considering the fact that reactive oxygen species, one of the crucial components of plasma, is able to change the methylation status in many cases [20, 21], it is reasonable to anticipate that plasma may also act on cells through the induction of epigenetic methylation changes.

The *LINE1* element comprises ~20% of the human genome (10^5^ copies/genome), is 6000–7000 bp long [22]. *Alu*, a short interspersed element (SINE), is a ~300 bp repetitive sequence and most abundant SINE, with ~10^6^ copies/genome, comprising 11% of the genome [23]. In normal tissues, both *LINE1* and *Alu* sequences are hypermethylated, however these elements can become hypomethylated in cancer [24, 25]. Hypomethylation of the two elements can also drive the expression of neighboring genes in cancer cells.

In this study, global, as well as genome-wide, methylation changes were monitored in two representative breast cancer cell types, estrogen-positive MCF-7 and estrogen-negative MDA-MB-231 cells, following plasma treatment. An ingenuity pathway analysis was performed with the genes of which methylation levels were significantly altered. In addition, cellular activity changes, including apoptosis and cell proliferation, were assessed upon treatment with plasma. To the best of the authors’ knowledge, this study is the first to examine the effects of plasma on the methylation level of cancer cells.

## Materials and Methods

### Cell culture and treatment with plasma

MCF-10A (normal breast), MCF-12A (normal breast), MCF-7 (breast cancer), and MDA-MB-231 (breast cancer) cell lines were purchased from the American Type Culture Collection (ATCC, Manassas, VA). HCT-15 (colon cancer) and NCI-H1299 (lung cancer) were purchased from the Korean Culture Type Collection (KCTC, Korea). MCF-10A and MCF-12A cells were grown in MEBM supplemented with MEGM Single Quots and cholera toxin (Lonza, Basel, Switzerland). MCF-7, MDA-MB-231, HCT-15, and NCI-H1299 cells were cultured in RPMI-1640 medium supplemented with 10% fetal bovine serum. The cultured cells grown to 50% confluence in 60-mm culture dishes were exposed to plasma for 30 sec 10 times in all experiment except for ROS detection wherein plasma was treated for 600 sec., at a 2.24 kV strength under argon gas using a DBD-type of plasma-producing device manufactured at the Plasma Bioscience Research Center (Kwangwoon University, Korea). The cells were then cultured for an additional 24 hr before harvest.

### Apoptosis and cell proliferation assay

Cells treated with plasma in a 60-mm dish were harvested using 0.05% trypsin-EDTA (Gibco-BRL, Carlsbad, CA) and washed with PBS. To analyze apoptosis, 1x10^5^ washed cells were treated with 5 μl of FITC Annexin V and 5 μl of propidium iodide (PI) using an FITC Annexin Apoptosis Detection Kit (BD Technologies, Franklin Lakes, NJ). To analyze proliferation, the washed cells were fixed in ice-cold 70% ethanol overnight. The cells were then treated with 50 μg/ml RNaseA (Sigma-Aldrich, St. Louis, MO) for 1 hr and stained with 50 μg/ml PI (Sigma-Aldrich) in the dark for 30 min at room temperature. Samples were analyzed using a FACS Canto II flow cytometer (BD Technologies) and read with a 488-nm laser. The cell proliferation index was calculated using the following formula: proliferation index = (S+G2+M)/(G0/G1+S+G2+M) x 100%, where each letter represents the number of cells at each cell cycle stage. To monitor cell proliferation using a Cell Counting Kit-8 (Dojindo, Japan), 2 × 10^3^ cells were plated onto a 96-well plate and cultured for 6 days followed by staining of the cells with WST-8. For colony formation assay, cells were seeded into 60-mm cell culture dishes with medium at a density of 1.3 × 10^4^ cells/dish. Following treatment with plasma, cells were cultured for two weeks, fixed with acetic acid/methanol (1:7), and stained with 1% crystal violet.

### Pyrosequencing

Pyrosequencing is a sequencing-by-synthesis method that quantitatively monitors the real-time incorporation of nucleotides through the enzymatic conversion of released pyrophosphate into a proportional light signal. After bisulfite treatment and PCR, the degree of methylation at each CpG position in a sequence is determined from the ratio of T and C [26]. One microgram of genomic DNA sample was sodium bisulfite-converted using the EZ DNA Methylation Kit (Zymo Research). The resulting DNA was used to determine the methylation of human *LINE1* and *Alu* using quantitative pyrosequencing, as described previously using the PyroMark ID (Qiagen, Valencia, CA) [27]. Non-CpG cytosines served as internal controls to verify efficient bisulfite DNA conversion. The degree of methylation was expressed as the percentage of methylated cytosines over the sum of methylated and unmethylated cytosines. Sequencing was performed on five samples independently treated with plasma, and assays were performed a minimum of two times per sample.

### Genome-wide methylation assay

Chromosomal DNA from cells grown in a 60-mm culture dish was isolated using the AllPrep DNA/RNA/miRNA Universal Kit (Qiagen) with a final elution in 50 μl of distilled water. Fifty ng of chromosomal DNA was applied to the Illumina Infinium Human Methylation 450 Beadchip to detect genome-wide methylation (> 485,000 CpG sites). A methylation index (β) was outputted for each site, which is a continuous variable ranging between 0 and 1 that represented the ratio of the intensity of the methylated-probe signal to the total locus signal intensity. A β value of 0 corresponds to no methylation, while a value of 1 corresponds to 100% methylation at the specific CpG locus measured. These values were then used to calculate a ratio of relative methylation between control and plasma-treated cells, with higher values corresponding to greater levels of methylation in plasma-treated relative to non-treated cells. All array data were uploaded to the Gene Expression Omnibus (GEO) database, and can be accessed via their website (http://www.ncbi.nlm.nih.gov/geo/; Series accession number GSE65087).

### Pathway analysis

Functional categorization and pathway construction for the gene pool obtained from the genes affected by plasma treatment were performed using the Ingenuity Pathway Analysis (IPA) software tool (Ingenuity Systems, Redwood City, CA). *P* values for individual networks were obtained by comparing the likelihood of obtaining the same number of transcripts or greater in a random gene set as were actually present in the input file (i.e., the set of genes differentially methylated in non-treated versus plasma-treated groups) using Fisher’s exact test, based on the hypergeometric distribution. The highest confidence functional network was designated as the top network.

### Real-time reverse transcription-polymerase chain reaction (RT-PCR)

Total RNA from cells grown in a 60-mm culture dish was isolated using the AllPrep DNA/RNA/miRNA Universal Kit (Qiagen) with a final elution in 50 μl of distilled water. Reverse transcription was conducted using 2 μg of total RNA with a reverse transcription kit (Toyobo, Japan). Gene expression was measured by real-time quantitative RT-PCR analysis using a Kapa SYBR Fast qPCR Kit (Kapa Biosystems, Woburn, MA) on an ABI 7300 instrument (Applied Biosystems, Foster City, CA). One microliter of cDNA was used for PCR, which was performed in duplicate. The primers used for the RT-PCR are listed in S1 Table. RNA quantity was normalized to GAPDH content, and gene expression was quantified according to the 2^-ΔCt^ method.

### ROS detection assay

Cells were cultured in 96-well plates at 4 × 10^3^ cells/well for 24 hr and treated with cold plasma for 600 sec. ROS generation was detected with DCFH-DA (Sigma-Aldrich) following the manufacturer's instructions. Briefly, after plasma treatment, cells were incubated with 1 mM of DCFH-DA for 30 min at 37°C in the dark. ROS were measured by an Infinite 200 Pro fluorescence plate reader (Tecan, Switzerland) at excitation and emission wavelengths of 485 and 535 nm, respectively. Assays were performed in triplicate.

### Statistical analysis

In the microarray analysis, observations with adjusted p-values equal to or greater than 0.05 were removed and thus precluded from further analysis. Following adjustment, remaining genes were defined as differentially methylated if they displayed an increased or decreased methylation level equal to or higher than 0.2 compared to control. A student’s t-test was used to detect differences in the mean levels of expression for selected genes. Statistical analyses were conducted using SPSS for Windows, release 17.0 (SPSS Inc., Chicago, IL). A p < 0.05 was considered to be statistically significant. The heatmap data were obtained using the Multiexperiment Viewer software (http://www.tm4.org/). A pool of genes that fit the top IPA network was submitted to the program.

## Results

### Plasma-induced global hypomethylation of Alu in MDA-MB-231 cells

**Fig 1:**
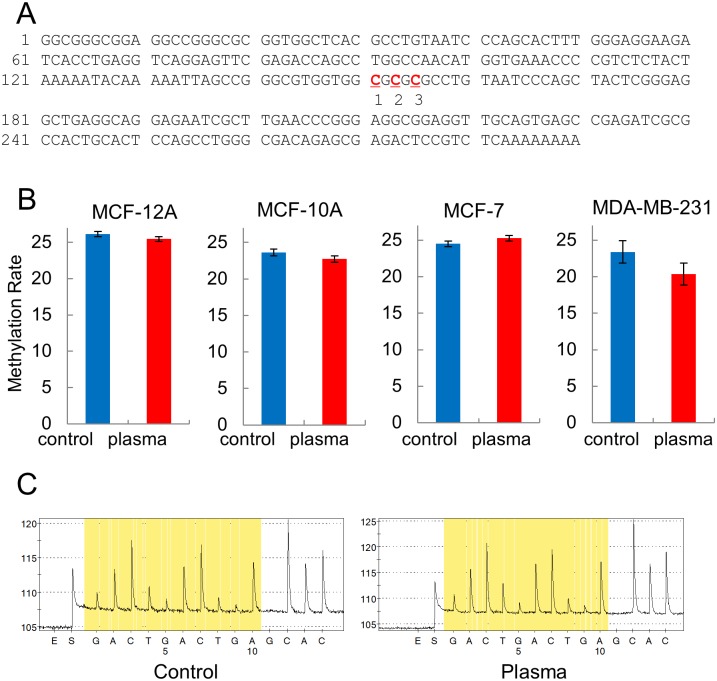
Effect of plasma on the global methylation levels in breast cell lines. The methylation levels of the four CpGs on the *Alu* from the MCF-12A, MCF-10A, MCF-7, and MDA-MB-231 cells were determined by pyrosequencing after treatment with plasma. (A) The sequence of the *Alu* adopted in this study [42]. The three CpG sites analyzed are indicated in red and numbered. (B) Bar graphs showing the methylation levels of CpG #2 of *Alu* in the four cell lines. Five independent experiments were performed for each cell line and average values are given with the standard errors. (C) A representative pyrosequencing diagram for MDA-MB-231 cells.

To examine the effect of plasma on the induction of epigenetic changes in breast cancer cells, cultured MCF-7 and MDA-MB-231 cells, representing estrogen-positive and estrogen-negative cancer cells, respectively, as well as the normal breast cell lines MCF-10A and MCF-12A, were treated with plasma. Two repetitive elements, *LINE1* and *Alu*, were selected to monitor the global methylation changes induced by plasma treatment (Fig 1A). The pyrosequencing analysis indicated that *Alu* was hypomethylated at a CpG site, decreasing from 23.4 to 20.3%, by plasma treatment in the estrogen-negative MDA-MB-231 cells (p < 0.05) (Fig 1B and 1C). Two other CpG sites examined showed no significant changes. Also, neither the estrogen-positive MCF-7 nor the normal cell lines showed any significant methylation changes at all the examined CpG sites. In contrast to *Alu*, *LINE1* did not show any significant methylation change throughout the CpG sites in the MDA-MB-231 cell (S1 Fig). These results indicate that the plasma acts in a cell type-specific, as well as a CpG site-specific, manner.

### Cell-to-cell signaling and cancer-related pathways are involved in the network of methylation-altered genes by plasma

**Fig 2:**
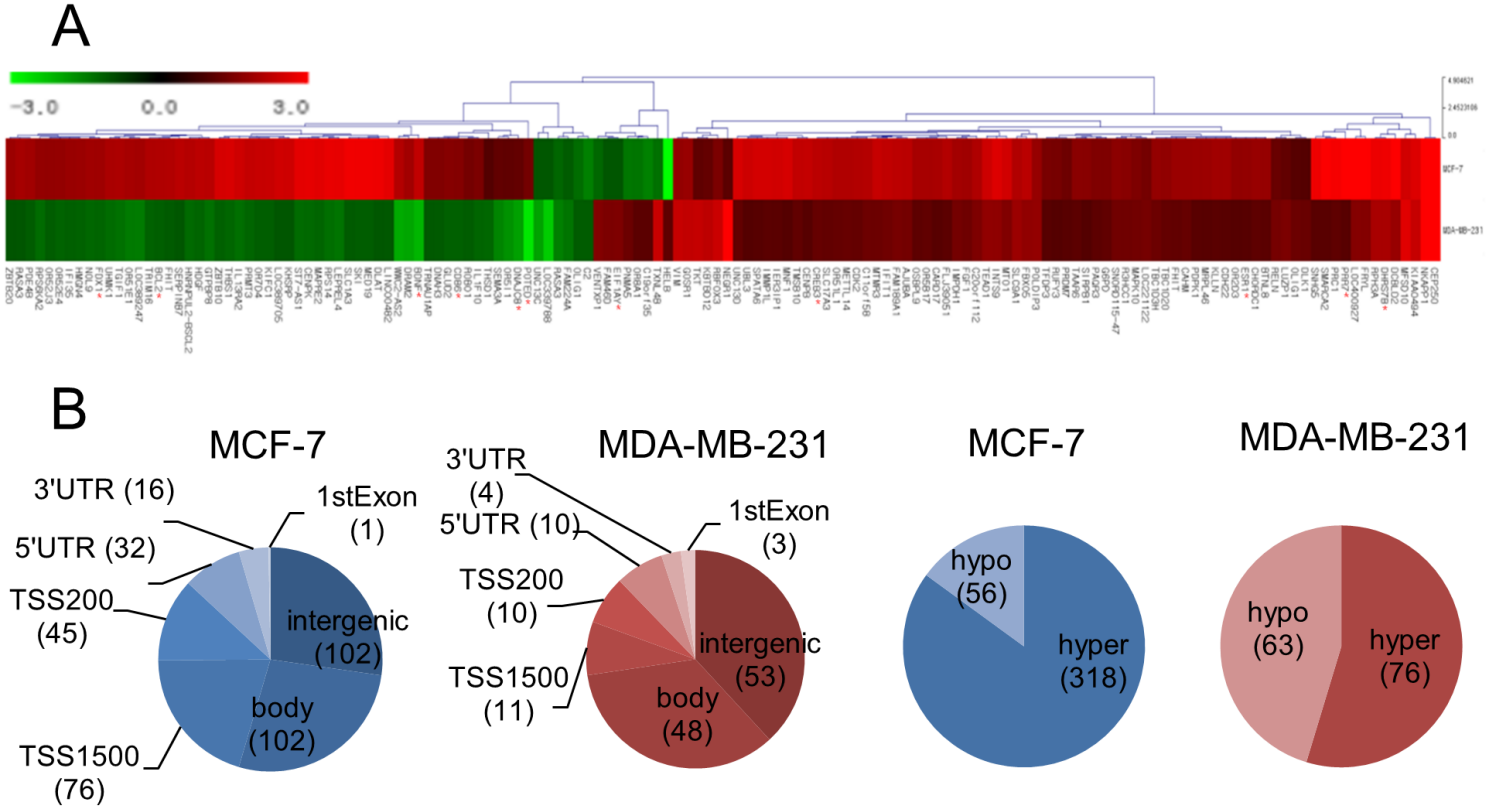
Genome-wide distributions of the CpG sites in which methylation levels were changed by plasma. The methylation levels of CpGs were analyzed on three 450k microarrays and the array data wherein the *Alu* methylation change is at the mean was adopted for further analysis. Genes showing significant changes by plasma compared to non-treated were presented. (A) Heatmap analysis of CpGs, showing significant, plasma-induced changes in methylation levels in the promoter region in the MCF-7 (upper row) and MDA-MB-231 (lower row) cancer cells. Genes showing hypermethylation and hypomethylation compared to control after plasma treatment were denoted in red and green, respectively. The number on the bar is a fold change of methylation. The genes marked with an asterisk are ones of which expression was examined by RT-PCR (Fig 5). (B) A Venn diagram showing the distribution of the CpGs on the chromosome and hyper- or hypomethylation in the MCF-7 and MDA-MB-231 cells after treatment with plasma. The number in parenthesis is the amount of the CpGs in each category.

**Fig 3:**
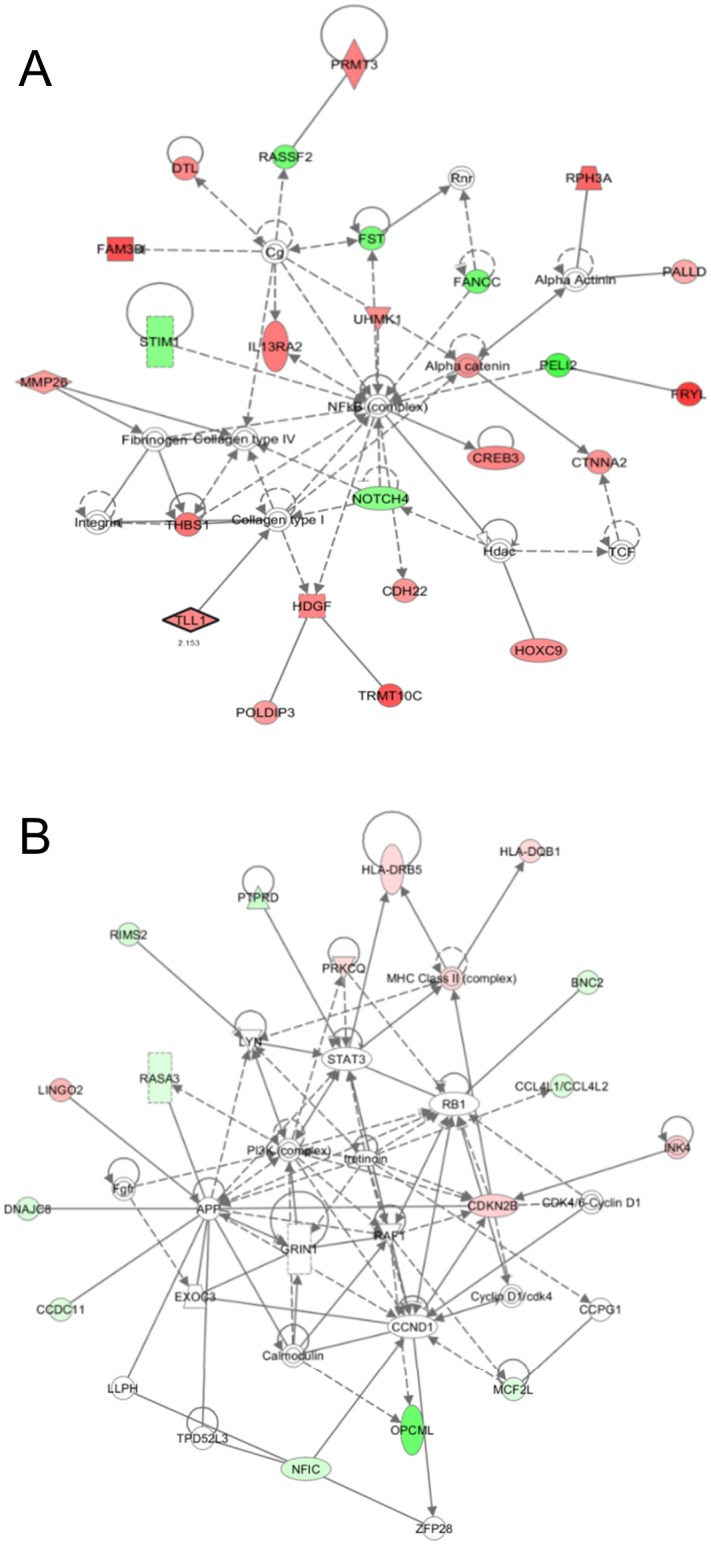
The highest confidence network of genes displaying altered methylation levels induced by plasma in the breast cancer cells. According to IPA, the highest confidence networks in the MCF-7 cells (A) and MDA-MB-231 cells (B) were “cellular movement, connective tissue development and function, tissue development” and “cell-to-cell signaling and interaction, cell death and survival, cellular development”, respectively. Genes that were hypermethylated in the plasma-treated cells are shaded in red, while those that were hypomethylated are shaded in green, with intensity signifying the magnitude of methylation change. Each interaction is supported by at least one literature reference, with solid lines representing direct interactions and dashed lines representing indirect interactions.

To address the effect of plasma on the epigenomic profiles of the MCF-7 and MDA-MB-231 cells, genome-wide methylation changes were monitored through microarray analysis. Genes fitting our criteria of methylation level changes (i.e., β-values higher than 0.2 after plasma treatment) were selected for further analysis. Among the selected genes, only RASA3 appeared in both cell types (Δβ: 0.23, MCF-7; -0.20, MDA-MB-231), although the involved CpG sites were located in different parts of the gene. In the MCF-7 cells, 318 and 56 CpG sites were hyper- and hypomethylated, respectively, in the plasma-treated cells (Fig 2). The CpGs appeared throughout the chromosome with 151 being found in the coding region, 121 in the promoter region, and 102 in the intergenic region (Fig 2B). The 374 CpG sites in the MCF-7 cells were examined for functional inter-relatedness using the Ingenuity Pathway Analysis software tool. The results indicated the “cellular movement, connective tissue development and function, tissue development” pathway as the top network and the “cancer”-involved pathway as the second top network, implying their roles in tumorigenesis (Fig 3A, S2 Fig, and Table 1). Of note, genes regulated by, or regulating, NF-κB featured most prominently in the network. The transcripts displaying the most significant levels of altered methylation within this network were IL13RA2 (hypermethylated, Δβ = 0.27) and PELI2 (hypomethylated, Δβ = -0.37). IL13RA2 expression has previously been established to be upregulated in lung-metastatic breast cancer cells [28] and in brain tumors [29]. PELI2 comprises one of several families of E3 ubiquitin ligases that play roles in regulating innate and adaptive immune receptor-signaling pathways [30].

**Table 1:** Genes in the top network displaying differential methylation in MCF-7 cells exposed to cold plasma.

Symbol	Accession	Description	Fold change
*CDH22*	NM_021248	Cadherin 22, type 2	1.90
*CREB3*	NM_006368	CAMP responsive element binding protein 3	2.22
*CTNNA2*	NM_004389	Catenin (cadherin-associated protein), alpha 2	1.96
*DTL*	NM_016448	Denticleless E3 ubiquitin protein ligase homolog (Drosophila)	2.14
*FAM3D*	NM_138805	Family with sequence similarity 3, member D	3.18
*FANCC*	NM_000136	Fanconi anemia, complementation group C	-1.56
*FRYL*	NM_015030	FRY-like	3.55
*FST*	NM_006350	Follistatin	-1.48
*HDGF*	NM_001126050	Hepatoma-derived growth factor	2.22
*HOXC9*	NM_006897	Homeobox C9	2.05
*IL13RA2*	NM_000640	Interleukin 13 receptor, alpha 2	2.44
*MMP26*	NM_021801	Matrix metallopeptidase 26	1.79
*NOTCH4*	NM_004557	Notch 4	-1.30
*PALLD*	NM_016081	Palladin, cytoskeletal associated protein	1.53
*PELI2*	NM_021255	Pellino E3 ubiquitin protein ligase family member 2	-2.00
*POLDIP3*	NM_032311	Polymerase (DNA-directed), delta interacting protein 3	1.78
*PRMT3*	NM_001145166	Protein arginine methyltransferase 3	2.32
*RASSF2*	NM_170774	Ras association (RalGDS/AF-6) domain family member 2	-1.53
*RPH3A*	NM_014954	Rabphilin 3A homolog (mouse)	2.80
*STIM1*	NM_003156	Stromal interaction molecule 1	-1.30
*THBS1*	NM_003246	Thrombospondin 1	2.56
*TLL1*	NM_012464	Thrombospondin 1	2.15
*TRMT10C*	NM_017819	TRNA methyltransferase 10 homolog C (S. cerevisiae)	3.05

In the MDA-MB-231 cells, 76 and 63 CpG sites were found to be hyper- and hypomethylated, respectively, following plasma treatment. A total of 65 CpGs were in the coding region, 21 in the promoter region, and 53 in the intergenic region. IPA analysis for the 139 genes identified the “cell-to-cell signaling and interaction, cell death and survival, cellular development” pathway as the top network (Fig 3B, S2 Fig, and Table 2). The transcripts displaying the most significant levels of altered methylation within this network were HLA-DQB1 (hypermethylated, Δβ = 0.21) and OPCML (hypomethylated, Δβ = -0.28). Upregulation of HLA-DQB1 has previously been reported in human esophageal squamous cell carcinoma [31] and in canine mammary tumor [32]. OPCML has a tumor-suppressor function in mammary and ovarian cancer, and epigenetic inactivation of the gene induces oncogenic transformation of ovarian surface epithelial cells [33, 34].

**Table 2:** Genes in the top network displaying differential methylation in MDA-MB-231 cells exposed to cold plasma.

Symbol	Accession	Description	Fold change
*BNC2*	NM_017637	Basonuclin 2	-1.62
*CCDC11*	NM_145020	Coiled-coil domain containing 11	-1.69
*CCL4L1/CCL4L2*	NM_001001435	Chemokine (C-C motif) ligand 4-like 1 / 2	-1.60
*CDKN2B*	NM_004936	Cyclin-dependent kinase inhibitor 2B (p15, inhibits CDK4)	1.73
*DNAJC8*	NM_014280	DnaJ (Hsp40) homolog, subfamily C, member 8	-1.89
*HLA-DQB1*	NM_002123	Major histocompatibility complex, class II, DQ beta 1	1.46
*HLA-DRB5*	NM_002125	Major histocompatibility complex, class II, DR beta 5	1.46
*LINGO2*	NM_001258282	Leucine rich repeat and Ig domain containing 2	2.64
*MCF2L*	NM_024979	MCF.2 cell line derived transforming sequence-like	-1.63
*NFIC*	NM_005597	Nuclear factor I/C (CCAAT-binding transcription factor)	-2.19
*OPCML*	NM_001012393	Opioid binding protein/cell adhesion molecule-like	-7.55
*PRKCQ*	NM_006257	Protein kinase C, theta	1.40
*PTPRD*	NM_130391	Protein tyrosine phosphatase, receptor type, D	-2.57
*RASA3*	NM_007368	RAS p21 protein activator 3	-1.51
*RIMS2*	NM_001100117	Regulating synaptic membrane exocytosis 2	-2.11

### Plasma induced an increase in apoptosis of the breast cancer cells

**Fig 4:**
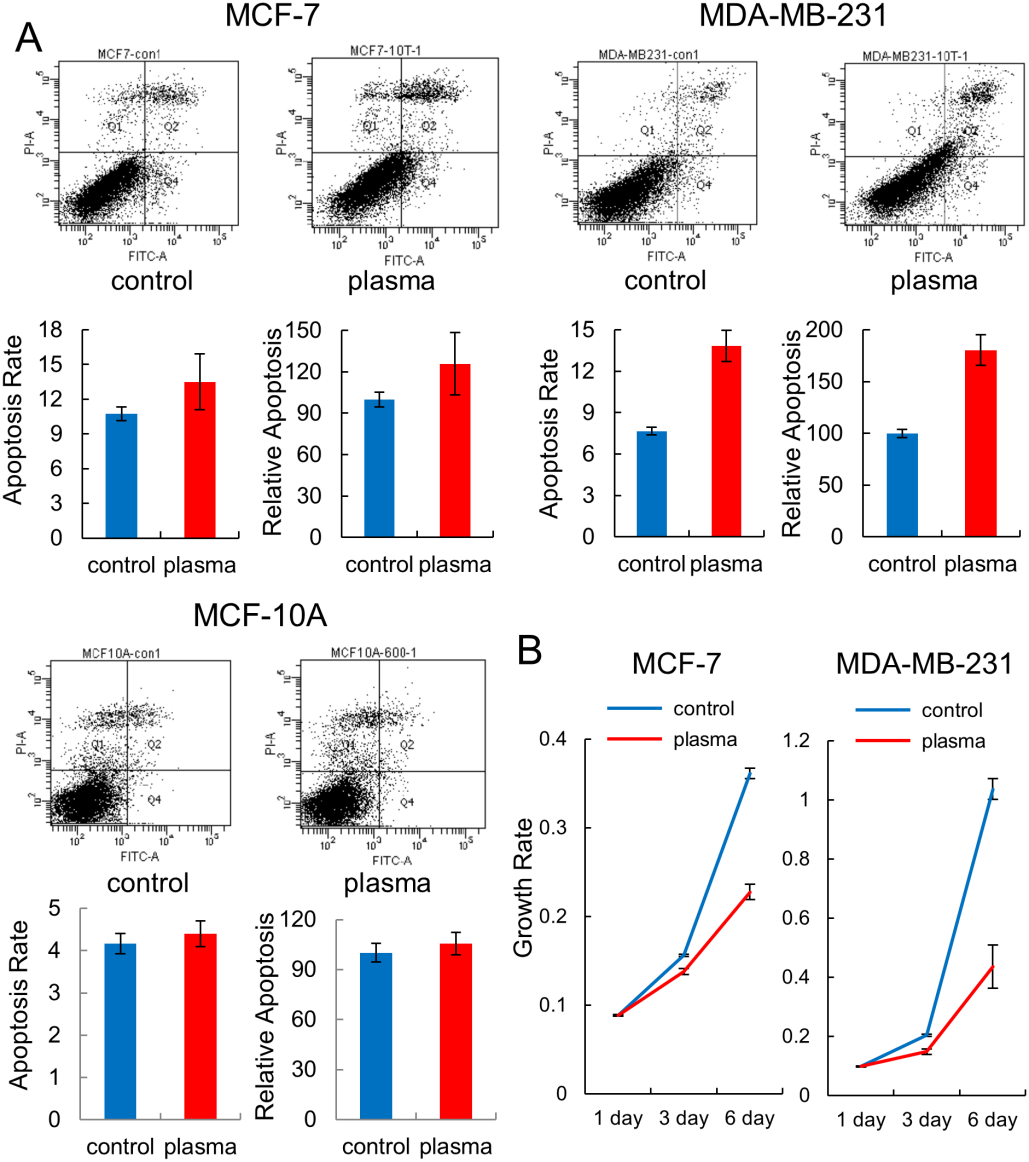
Apoptosis and cell proliferation assays on the breast cancer cells exposed to plasma. (A) MCF-7, MDA-MB-231, and MCF-10A cells were treated with plasma for 10 times (30 sec each time with an hour interval between exposures), and apoptosis was analyzed by FACS. The assay was performed in triplicate and the result is given by a representative FACS diagram. The ratio of cells undergoing apoptosis is denoted by a bar graph with average and standard errors. (B) Results of the cell proliferation assay by using Cell Counting Kit-8. Each sample was analyzed in triplicate, with the average growth rate and standard errors.

Based on the observation that plasma affected the methylation status of many genes involved in the “cell cycle”, “cell-to-cell signaling and interaction”, or “apoptosis pathway”, the apoptosis and proliferation of the breast cancer and normal cell lines were examined by FACS analysis after treatment with plasma. For apoptosis, an increase in apoptosis was observed in both cancer cell lines following treatment with plasma, but the increased rate was higher in the estrogen-negative MDA-MB-231 cells (from 7.67 to 13.8%) than in the estrogen-positive MCF-7 cells (from 10.7 to 13.5%) (Fig 4A). Meanwhile, no significant change was observed in the MCF-10A cell. The apoptotic effect of plasma was further examined in two more cancer cell lines other than breast cancer. The lung cancer cell line, NCI-H1299, showed a high increase of apoptosis (from 7.13 to 10.2%) while the colon cancer cell line, HCT-15, showed a less significant increase (from 8.53 to 9.16%) by plasma (S3 Fig).

The effect of plasma on cell proliferation was examined using a cell proliferation assay kit as well as through colony forming assay and the results indicated that the least number of cells were observed in the plasma-treated MDA-MB-231 cell, confirming that this cell line is most vulnerable to plasma regarding apoptosis (Fig 4B and S4 Fig). All these results indicate that plasma induces epigenetic and cell activity changes in a cell type-specific manner, inducing hypomethylation of *Alu* and increasing cellular apoptosis, especially in the MDA-MB-231 cells.

### Expression confirmation of the differentially methylated genes

**Fig 5:**
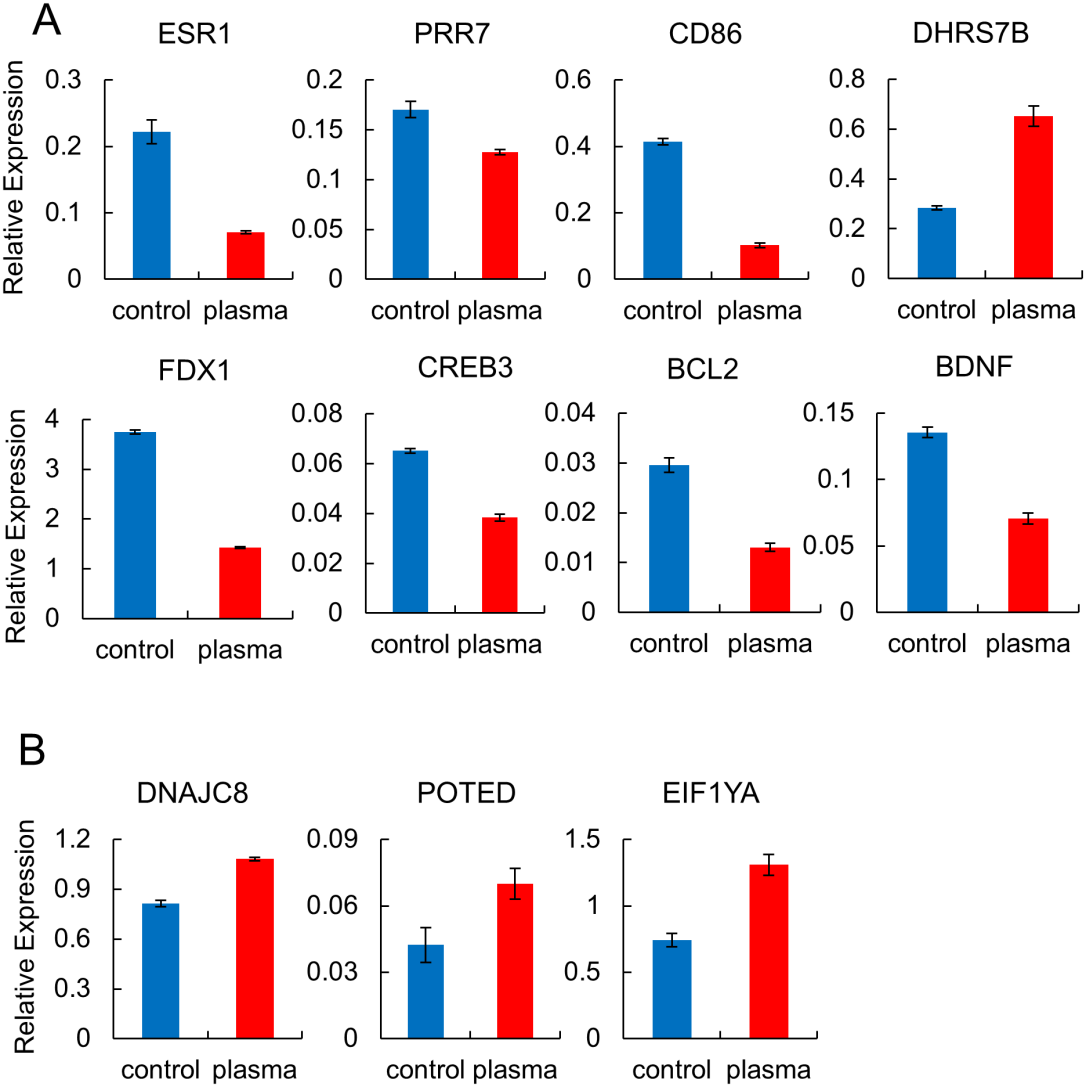
RT-PCR analysis of selected genes showing altered methylation after plasma treatment in breast cancer cells. Real-time RT-PCR analysis of selected genes displaying altered methylation levels in response to plasma in the MCF-7 (A) and MDA-MB-231 cells (B). Cells were treated 10 times, 30 sec each time with an hour interval between exposures, and then harvested after 24 hr. Each sample was analyzed in triplicate, with the average relative expression levels indicated with standard errors.

Expression of selected hypermethylated or hypomethylated genes was determined by real-time RT-PCR for the plasma-treated MCF-7 and MDA-MB-231 cells used in the genome-wide methylation assay to check for consistency between mRNA levels and DNA methylation. 45 and 10 CpGs appeared within 200 bp upstream of the transcription start site in the plasma-treated MCF-7 and MDA-MB-231 cell, respectively (Fig 2B). Nine hypermethylated genes (ESR1, PRR7, CD86, FDX1, CREB3, DHRS7B, EIF1YA, BCL2, and BDNF) and two hypomethylated genes (DNAJC8 and POTED) were selected, of which CpG sites were located in the promoter region. As shown in Fig 5, all the genes showed up- or downregulation in accordance with the methylation status except for DHRS7B and EIF1YA, which were upregulated while the CpGs were hypermethylated.

## Discussion

Altered methylation of *Alu* has been implicated in various cancer types, which may cause genomic instability, eventually leading to cancer of the cell. Across the four cell types examined in this study, only one specific CpG site in the estrogen-negative cancer cell line, MDA-MB-231, showed a significant change in methylation levels, being hypomethylated by the plasma treatment. The hypomethylation observed in this study is not considered to drive the cell into a more cancerous state because the plasma-treated cell showed anti-proliferation activities, as evidenced by the cell proliferation and apoptosis assays and the IPA pathway analysis. Specifically, among the MCF-10A, MCF-12A, MCF-7, and MDA-MB-231 cell lines, the MDA-MB-231 cells showed a remarkable increase of apoptosis by the plasma treatment. Also, it is notable that a significant methylation change in *Alu* was only observed in the MDA-MB-231 cells. The possibility that the other cell types were not properly exposed to the plasma can be excluded, as genome-wide methylation changes were observed through the methylation chip analysis. Assuring sufficient exposure to the target cells is a crucial step before further examining the effect of plasma. A few molecular markers and cellular activities, including the production of hydrogen peroxide [2] and induction of apoptosis [35], are currently proposed. *Alu* can be used as an epigenetic marker, at least in limited cancer cells, including MDA-MB-231 cells.

**Fig 6:**
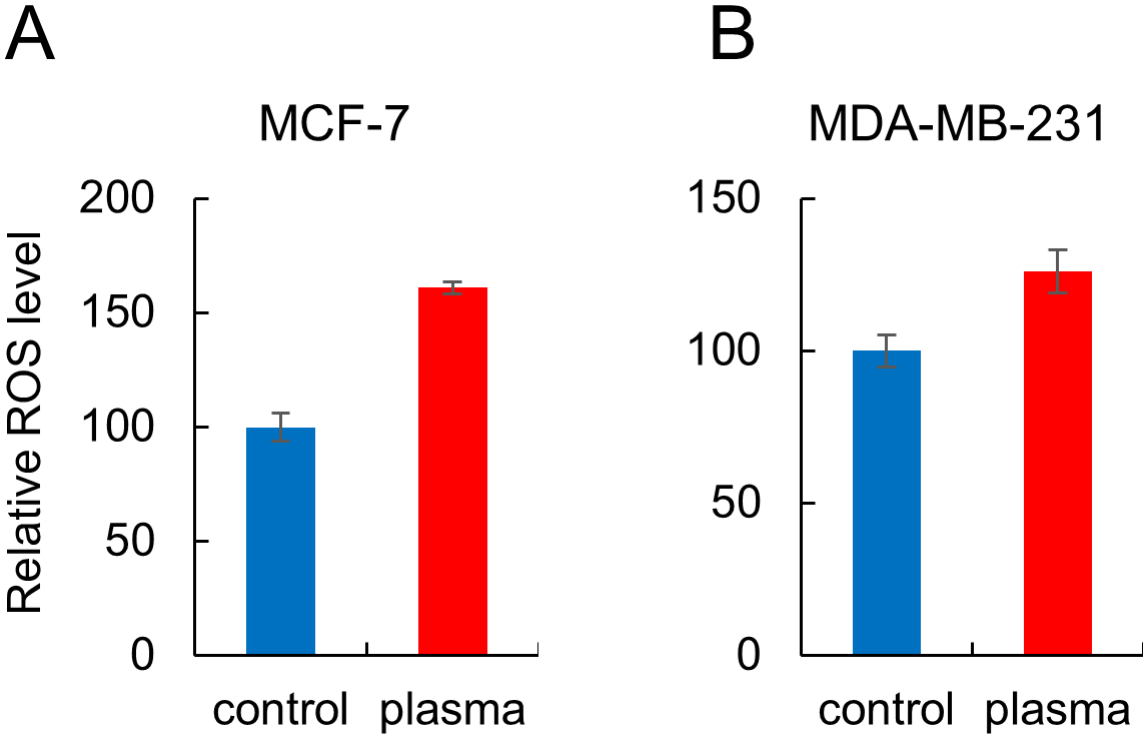
Cold plasma enhances ROS accumulation in breast cancer cells. Cells were treated with cold plasma for 600 sec. ROS level was quantified by the fluorescent dye DCFH-DA and is shown as a fold change relative to non-treated cells. Each sample was analyzed in triplicate, with the average relative levels indicated with standard errors.

It seems that the plasma treatment utilized in this study did not induce wide-range methylation changes in terms of the number of affected sites because only 121 and 21 CpGs in the promoter region in MCF-7 and MDA-MB-231 cells showed significant changes. Meanwhile, other physical sources, such as γ-rays, have been reported to alter 442 CpGs in the promoter region in human bronchial epithelial cells [36]. In a previous study, ROS have been reported to relay signals inducing methylation changes at the CpG sites in a number of genes, including BCL-2, BDNF, p16, RUNX3, and E-cadherin [20]. Especially, BCL-2 (Δβ: 0.23) and BDNF (Δβ: 0.21) also appeared on the list of genes that have shown a significant methylation change by plasma treatment in the MCF-7 cells. Expression of the two genes was inversely correlated with their methylation status in the plasma-treated group, judged by RT-PCR (Fig 5A). We also observed an increase of ROS level in the MCF-7 and MDA-MB-231 cell by plasma treatment (Fig 6). Taken together, these results confirm that plasma provokes changes in cellular activities via ROS and eventually regulates gene activity through alteration of CpG methylation status. BCL-2 is an anti-apoptotic protein, originally identified as an oncogene at the chromosomal breakpoint of t(14;18) involved in human follicular B cell lymphomas [37]. BDNF has a critical role in tumorigenesis, promoting proliferation, differentiation, angiogenesis and invasiveness in several tumor types, including breast cancer [38]. It is notable that these genes were hypermethylated by plasma, possibly suppressing their oncogenic activities.

It should be mentioned that plasma and ROS shared little in common, as the majority of the genes altered by methylation appeared unique to each stimulus. The IPA pathway analysis revealed the “TGF-β Signaling” and “Calcium-induced T Lymphocyte Apoptosis” pathways as the highest probable canonical pathways in MCF-7 and MDA-MB-231 cells, respectively. TGF1, HoxC9, HLA-DQB1, and PRKCQ are representative genes involved in the pathways, showing altered methylation by plasma. In contrast, previous studies identified ATM-dependent signaling [39] and ETM pathway [40] as ROS-linked pathways implying that plasma can also act on the cell by routes other than those of ROS.

A key limitation of our study design is the inability to assess methylation levels with respect to diverse plasma-treatment conditions. Thus far, information regarding both the types of plasma sources and applied exposure conditions is sparse, as the use of plasma treatment is still under development. In particular, choosing the appropriate exposure time of plasma treatment is crucial in observing the effects of plasma, as changing the strength or duration of plasma treatment causes different results. For example, HeLa and adipose-derived stem cells exhibited increased expression of apoptotic markers, including cleaved caspase-3, PARP, and phospho-p53 in proportion to the duration of the plasma treatment [14]. ROS, one of the mediators of plasma, also induces opposing cellular activities depending on its concentration [41]. Whereas low concentrations of ROS generally stimulate proliferation, high concentrations result in cell death. Low concentrations of ROS activated phosphorylation of ERKs, whereas high concentrations of ROS activated phosphorylation of JNKs. MLK3 directly phosphorylates JNKs and may control activation of ERKs [41]. Also in our studies, two independent plasma treatment conditions with different treatment schemes (one for a one-time 600 sec exposure and the other for 30 sec exposures a total of 10 times) caused changes in different cellular activities, with the latter causing increased cellular apoptosis (data not shown).

In summary, we present global, as well as genome-wide, methylation profiles of plasma-treated breast cancer cells, which we compiled using the most comprehensive methylation measurement techniques currently available. Also, the effects of plasma on cellular proliferation and apoptosis were presented. Our findings support the hypothesis that epigenetic dysregulation of crucial cancer-relevant molecules, including those pertinent to tumor development and apoptosis, may be involved in the effects of plasma. Further investigations into the mechanism(s) leading to differential methylation observed in this study, as well as the association of methylation status with cellular activities, may provide additional insights into the epigenetic role of plasma, along with determining its treatment options for cancerous cells and tissues.

## Supporting Information

S1 Fig Effect of plasma on the *LINE1* methylation levels in the MDA-MB-231 cell. The methylation levels of the four CpGs on the *LINE1* from the MDA-MB-231 cell were determined by pyrosequencing after treatment with plasma. (A) The sequence of the *LINE1* adopted in this study. The four CpG sites analyzed are indicated in red and numbered. (B) Bar graphs showing the methylation levels of CpGs of *LINE1* in the MDA-MB-231. Five independent experiments were performed for each CpG and average values are given with the standard errors.
(DOCX)

S2 Fig Pathways most strongly associated with the significantly altered genes in the cold plasma-treated cells. Top functional categories are given for altered genes in the MCF-7 (A) and MDA-MB-231 cells (B). The Ingenuity software assigns a *p*-value based on the likelihood of obtaining the observed number of category- or pathway-related molecules in a given dataset by chance alone. The threshold line denotes the *p* = 0.05 level. The line graph represents the ratio of affected genes to the total number of genes in a pathway.
(DOCX)

S3 Fig Apoptosis assay of the colon and lung cancer cell line exposed to plasma. HCT-15 (colon) (A) and NCI-H1299 (lung) (B) cancer cells were treated with plasma for 10 times (30 sec each time with an hour interval between exposures), and apoptosis was analyzed by FACS. The assay was performed in triplicate and the result is given by a representative FACS diagram. The ratio of cells undergoing apoptosis is denoted by a bar graph with average and standard errors.
(DOCX)

S4 Fig Anti-proliferation effect of plasma on the breast cancer cells. MCF-7 (A) and MDA-MB-231 cells (B) were treated with plasma and the cell proliferation assay was carried out through colony forming assay. The top and bottom row of each panel are results for plasma non-treated and treated cells, respectively.
(DOCX)

S1 Table Sequences of primers employed in this study. (DOCX)

## References

[1] Wang M, Holmes B, Cheng X, Zhu W, Keidar M, Zhang LG. Cold atmospheric plasma for selectively ablating metastatic breast cancer cells. PloS one. 2013;8(9):e73741 10.1371/journal.pone.0073741 24040051 PMC3770688

[2] Bekeschus S, Kolata J, Winterbourn C, Kramer A, Turner R, Weltmann KD, et al Hydrogen peroxide: A central player in physical plasma-induced oxidative stress in human blood cells. Free radical research. 2014;48(5):542–9. 10.3109/10715762.2014.892937 . 24528134

[3] Liebmann J, Scherer J, Bibinov N, Rajasekaran P, Kovacs R, Gesche R, et al Biological effects of nitric oxide generated by an atmospheric pressure gas-plasma on human skin cells. Nitric oxide: biology and chemistry / official journal of the Nitric Oxide Society. 2011;24(1):8–16. 10.1016/j.niox.2010.09.005 . 20883806

[4] Keidar M, Walk R, Shashurin A, Srinivasan P, Sandler A, Dasgupta S, et al Cold plasma selectivity and the possibility of a paradigm shift in cancer therapy. British journal of cancer. 2011;105(9):1295–301. 10.1038/bjc.2011.386 21979421 PMC3241555

[5] Kim JY, Ballato J, Foy P, Hawkins T, Wei Y, Li J, et al Single-cell-level cancer therapy using a hollow optical fiber-based microplasma. Small. 2010;6(14):1474–8. 10.1002/smll.201000480 . 20586057

[6] Ishaq M, Evans MM, Ostrikov KK. Effect of atmospheric gas plasmas on cancer cell signaling. International journal of cancer Journal international du cancer. 2014;134(7):1517–28. 10.1002/ijc.28323 . 23754175

[7] Utsumi F, Kajiyama H, Nakamura K, Tanaka H, Mizuno M, Ishikawa K, et al Effect of indirect nonequilibrium atmospheric pressure plasma on anti-proliferative activity against chronic chemo-resistant ovarian cancer cells in vitro and in vivo. PloS one. 2013;8(12):e81576 10.1371/journal.pone.0081576 24367486 PMC3867316

[8] Guerrero-Preston R, Ogawa T, Uemura M, Shumulinsky G, Valle BL, Pirini F, et al Cold atmospheric plasma treatment selectively targets head and neck squamous cell carcinoma cells. International journal of molecular medicine. 2014;34(4):941–6. 10.3892/ijmm.2014.1849 25050490 PMC4152136

[9] Koritzer J, Boxhammer V, Schafer A, Shimizu T, Klampfl TG, Li YF, et al Restoration of sensitivity in chemo-resistant glioma cells by cold atmospheric plasma. PloS one. 2013;8(5):e64498 10.1371/journal.pone.0064498 23704990 PMC3660344

[10] Arndt S, Wacker E, Li YF, Shimizu T, Thomas HM, Morfill GE, et al Cold atmospheric plasma, a new strategy to induce senescence in melanoma cells. Experimental dermatology. 2013;22(4):284–9. 10.1111/exd.12127 . 23528215

[11] Partecke LI, Evert K, Haugk J, Doering F, Normann L, Diedrich S, et al Tissue tolerable plasma (TTP) induces apoptosis in pancreatic cancer cells in vitro and in vivo. BMC cancer. 2012;12:473 10.1186/1471-2407-12-473 23066891 PMC3598726

[12] Chang JW, Kang SU, Shin YS, Kim KI, Seo SJ, Yang SS, et al Non-thermal atmospheric pressure plasma induces apoptosis in oral cavity squamous cell carcinoma: Involvement of DNA-damage-triggering sub-G(1) arrest via the ATM/p53 pathway. Archives of biochemistry and biophysics. 2014;545:133–40. 10.1016/j.abb.2014.01.022 . 24486404

[13] Arndt S, Unger P, Wacker E, Shimizu T, Heinlin J, Li YF, et al Cold atmospheric plasma (CAP) changes gene expression of key molecules of the wound healing machinery and improves wound healing in vitro and in vivo. PloS one. 2013;8(11):e79325 10.1371/journal.pone.0079325 24265766 PMC3825691

[14] Ma Y, Ha CS, Hwang SW, Lee HJ, Kim GC, Lee KW, et al Non-thermal atmospheric pressure plasma preferentially induces apoptosis in p53-mutated cancer cells by activating ROS stress-response pathways. PloS one. 2014;9(4):e91947 10.1371/journal.pone.0091947 24759730 PMC3997341

[15] Yan PS, Shi H, Rahmatpanah F, Hsiau TH, Hsiau AH, Leu YW, et al Differential distribution of DNA methylation within the RASSF1A CpG island in breast cancer. Cancer research. 2003;63(19):6178–86. . 14559801

[16] Faryna M, Konermann C, Aulmann S, Bermejo JL, Brugger M, Diederichs S, et al Genome-wide methylation screen in low-grade breast cancer identifies novel epigenetically altered genes as potential biomarkers for tumor diagnosis. FASEB journal: official publication of the Federation of American Societies for Experimental Biology. 2012;26(12):4937–50. 10.1096/fj.12-209502 . 22930747

[17] Aypar U, Morgan WF, Baulch JE. Radiation-induced epigenetic alterations after low and high LET irradiations. Mutation research. 2011;707(1–2):24–33. 10.1016/j.mrfmmm.2010.12.003 . 21159317

[18] Ye S, Yuan D, Xie Y, Pan Y, Shao C. Role of DNA methylation in the adaptive responses induced in a human B lymphoblast cell line by long-term low-dose exposures to gamma-rays and cadmium. Mutation research Genetic toxicology and environmental mutagenesis. 2014;773:34–8. 10.1016/j.mrgentox.2014.08.004 . 25308704

[19] Goetz W, Morgan MN, Baulch JE. The effect of radiation quality on genomic DNA methylation profiles in irradiated human cell lines. Radiation research. 2011;175(5):575–87. 10.1667/RR2390.1 . 21375360

[20] Wu Q, Ni X. ROS-Mediated DNA Methylation Pattern Alterations in Carcinogenesis. Current drug targets. 2015;16(1):13–9. . 25585126 10.2174/1389450116666150113121054

[21] Jha P, Pia Patric IR, Shukla S, Pathak P, Pal J, Sharma V, et al Genome-wide methylation profiling identifies an essential role of reactive oxygen species in pediatric glioblastoma multiforme and validates a methylome specific for H3 histone family 3A with absence of G-CIMP/isocitrate dehydrogenase 1 mutation. Neuro-oncology. 2014;16(12):1607–17. 10.1093/neuonc/nou113 24997139 PMC4232083

[22] Pickeral OK, Makalowski W, Boguski MS, Boeke JD. Frequent human genomic DNA transduction driven by LINE-1 retrotransposition. Genome research. 2000;10(4):411–5. 10779482 10.1101/gr.10.4.411 PMC310862

[23] Lander ES, Linton LM, Birren B, Nusbaum C, Zody MC, Baldwin J, et al Initial sequencing and analysis of the human genome. Nature. 2001;409(6822):860–921. 10.1038/35057062 . 11237011

[24] Chalitchagorn K, Shuangshoti S, Hourpai N, Kongruttanachok N, Tangkijvanich P, Thong-ngam D, et al Distinctive pattern of LINE-1 methylation level in normal tissues and the association with carcinogenesis. Oncogene. 2004;23(54):8841–6. 10.1038/sj.onc.1208137 . 15480421

[25] Kochanek S, Renz D, Doerfler W. DNA methylation in the Alu sequences of diploid and haploid primary human cells. The EMBO journal. 1993;12(3):1141–51. 8384552 10.1002/j.1460-2075.1993.tb05755.x PMC413315

[26] Tost J, Gut IG. DNA methylation analysis by pyrosequencing. Nature protocols. 2007;2(9):2265–75. 10.1038/nprot.2007.314 . 17853883

[27] Woloszynska-Read A, Mhawech-Fauceglia P, Yu J, Odunsi K, Karpf AR. Intertumor and intratumor NY-ESO-1 expression heterogeneity is associated with promoter-specific and global DNA methylation status in ovarian cancer. Clinical cancer research: an official journal of the American Association for Cancer Research. 2008;14(11):3283–90. 10.1158/1078-0432.CCR-07-5279 18519754 PMC2835568

[28] Minn AJ, Gupta GP, Siegel PM, Bos PD, Shu W, Giri DD, et al Genes that mediate breast cancer metastasis to lung. Nature. 2005;436(7050):518–24. 10.1038/nature03799 16049480 PMC1283098

[29] Saka M, Amano T, Kajiwara K, Yoshikawa K, Ideguchi M, Nomura S, et al Vaccine therapy with dendritic cells transfected with Il13ra2 mRNA for glioma in mice. Journal of neurosurgery. 2010;113(2):270–9. 10.3171/2009.9.JNS09708 . 19895199

[30] Huoh YS, Ferguson KM. The pellino e3 ubiquitin ligases recognize specific phosphothreonine motifs and have distinct substrate specificities. Biochemistry. 2014;53(30):4946–55. 10.1021/bi5005156 25027698 PMC4201300

[31] Hu JM, Li L, Chen YZ, Liu C, Cui X, Yin L, et al HLA-DRB1 and HLA-DQB1 methylation changes promote the occurrence and progression of Kazakh ESCC. Epigenetics: official journal of the DNA Methylation Society. 2014;9(10):1366–73. 10.4161/15592294.2014.969625 . 25437052 PMC4623353

[32] Pawlowski KM, Homa A, Bulkowska M, Majchrzak K, Motyl T, Krol M. Expression of inflammation-mediated cluster of genes as a new marker of canine mammary malignancy. Veterinary research communications. 2013;37(2):123–31. 10.1007/s11259-013-9554-1 23435839 PMC3646156

[33] Cui Y, Ying Y, van Hasselt A, Ng KM, Yu J, Zhang Q, et al OPCML is a broad tumor suppressor for multiple carcinomas and lymphomas with frequently epigenetic inactivation. PloS one. 2008;3(8):e2990 10.1371/journal.pone.0002990 18714356 PMC2500176

[34] Zhou F, Tao G, Chen X, Xie W, Liu M, Cao X. Methylation of OPCML promoter in ovarian cancer tissues predicts poor patient survival. Clinical chemistry and laboratory medicine: CCLM / FESCC. 2014;52(5):735–42. 10.1515/cclm-2013-0736 . 24327526

[35] Thiyagarajan M, Anderson H, Gonzales XF. Induction of apoptosis in human myeloid leukemia cells by remote exposure of resistive barrier cold plasma. Biotechnology and bioengineering. 2014;111(3):565–74. 10.1002/bit.25114 . 24022746

[36] Lahtz C, Bates SE, Jiang Y, Li AX, Wu X, Hahn MA, et al Gamma irradiation does not induce detectable changes in DNA methylation directly following exposure of human cells. PloS one. 2012;7(9):e44858 10.1371/journal.pone.0044858 23024770 PMC3443085

[37] Tsujimoto Y, Croce CM. Analysis of the structure, transcripts, and protein products of bcl-2, the gene involved in human follicular lymphoma. Proceedings of the National Academy of Sciences of the United States of America. 1986;83(14):5214–8. 3523487 10.1073/pnas.83.14.5214 PMC323921

[38] Geiger TR, Peeper DS. Critical role for TrkB kinase function in anoikis suppression, tumorigenesis, and metastasis. Cancer research. 2007;67(13):6221–9. 10.1158/0008-5472.CAN-07-0121 . 17616679

[39] Alpay M, Backman LR, Cheng X, Dukel M, Kim WJ, Ai L, et al Oxidative stress shapes breast cancer phenotype through chronic activation of ATM-dependent signaling. Breast cancer research and treatment. 2015;151(1):75–87. 10.1007/s10549-015-3368-5 . 25862169

[40] Deferme L, Briede JJ, Claessen SM, Cavill R, Kleinjans JC. Cell line-specific oxidative stress in cellular toxicity: A toxicogenomics-based comparison between liver and colon cell models. Toxicology in vitro: an international journal published in association with BIBRA. 2015;29(5):845–55. 10.1016/j.tiv.2015.03.007 . 25800948

[41] Lee HS, Hwang CY, Shin SY, Kwon KS, Cho KH. MLK3 is part of a feedback mechanism that regulates different cellular responses to reactive oxygen species. Science signaling. 2014;7(328):ra52 10.1126/scisignal.2005260 . 24894995

[42] Kile ML, Baccarelli A, Tarantini L, Hoffman E, Wright RO, Christiani DC. Correlation of global and gene-specific DNA methylation in maternal-infant pairs. PloS one. 2010;5(10):e13730 10.1371/journal.pone.0013730 21060777 PMC2966409

