# Supplementary figures and images for: Differential Epigenetic Effects of Atmospheric Cold Plasma on MCF-7 and MDA-MB-231 Breast Cancer Cells
Source: PLoS One. 2015 Jun 4;10(6):e0129931. doi: 10.1371/journal.pone.0129931 (PMC4456358; doi:10.1371/journal.pone.0129931)

**S1 Fig. Effect of plasma on the *LINE1* methylation levels in the MDA-MB-231 cell.**

A


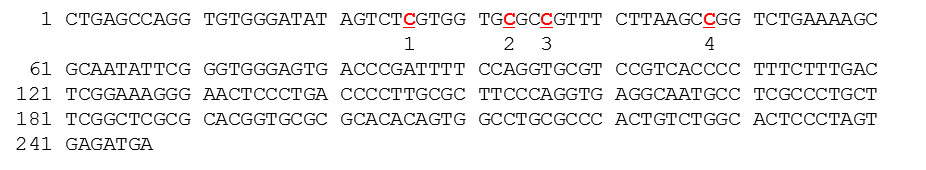


B

**
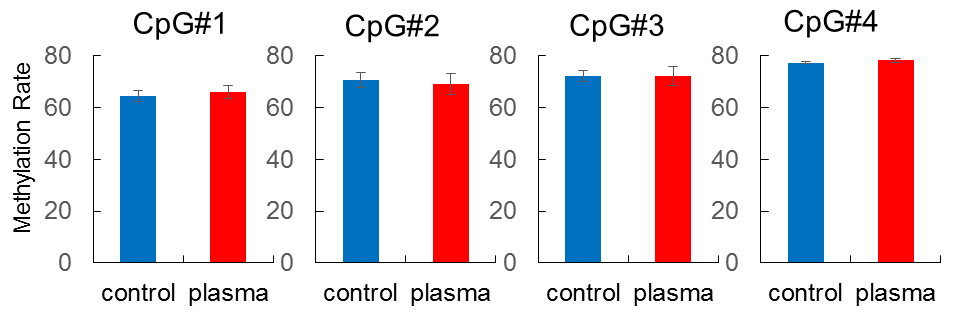
**

Supplement: S1 Fig — The methylation levels of the four CpGs on the LINE1 from the MDA-MB-231 cell were determined by pyrosequencing after treatment with plasma. (A) The sequence of the LINE1 adopted in this study. The four CpG sites analyzed are indicated in red and numbered. (B) Bar graphs showing the methylation levels of CpGs of LINE1 in the MDA-MB-231. Five independent experiments were performed for each CpG and average values are given with the standard errors. (DOCX) [file pone.0129931.s001.docx]

**S3 Fig. Apoptosis assay of the colon and lung cancer cell line exposed to plasma.**


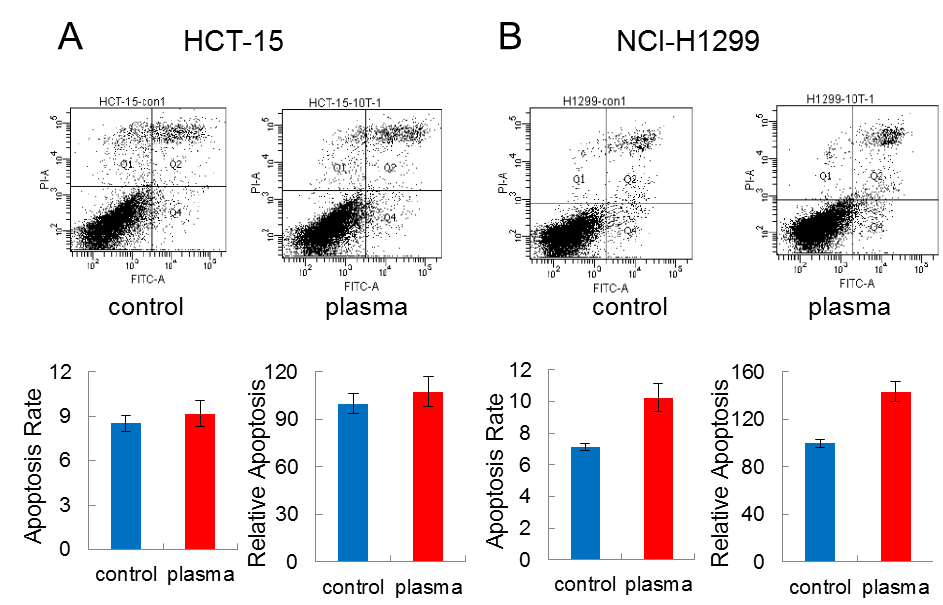

Supplement: S3 Fig — HCT-15 (colon) (A) and NCI-H1299 (lung) (B) cancer cells were treated with plasma for 10 times (30 sec each time with an hour interval between exposures), and apoptosis was analyzed by FACS. The assay was performed in triplicate and the result is given by a representative FACS diagram. The ratio of cells undergoing apoptosis is denoted by a bar graph with average and standard errors. (DOCX) [file pone.0129931.s003.docx]

**S4 Fig. Anti-proliferation effect of plasma on the breast cancer cells.**

**
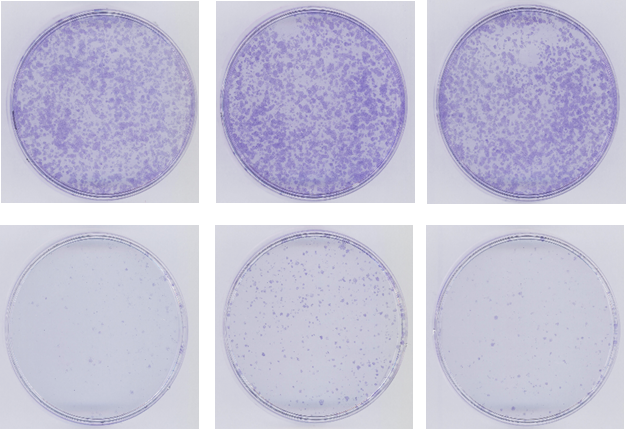

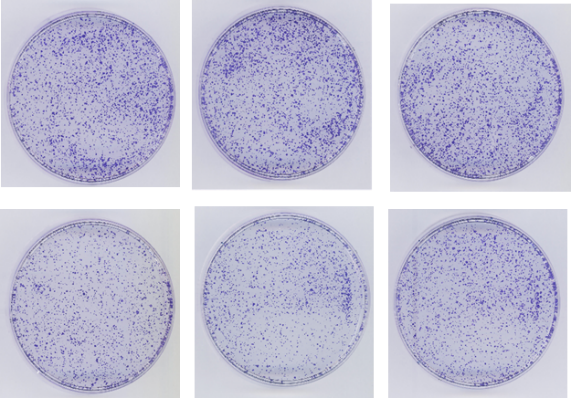
**

B

A

Supplement: S4 Fig — MCF-7 (A) and MDA-MB-231 cells (B) were treated with plasma and the cell proliferation assay was carried out through colony forming assay. The top and bottom row of each panel are results for plasma non-treated and treated cells, respectively. (DOCX) [file pone.0129931.s004.docx]
