# Supplementary material for: Differential Epigenetic Effects of Atmospheric Cold Plasma on MCF-7 and MDA-MB-231 Breast Cancer Cells
Source: PLoS One. 2015 Jun 4;10(6):e0129931. doi: 10.1371/journal.pone.0129931 (PMC4456358; doi:10.1371/journal.pone.0129931)
Supplement: S2 Fig — Top functional categories are given for altered genes in the MCF-7 (A) and MDA-MB-231 cells (B). The Ingenuity software assigns a p-value based on the likelihood of obtaining the observed number of category- or pathway-related molecules in a given dataset by chance alone. The threshold line denotes the p = 0.05 level. The line graph represents the ratio of affected genes to the total number of genes in a pathway. (DOCX) [file pone.0129931.s002.docx]

**S2 Fig. Pathways most strongly associated with the significantly altered genes in the cold plasma-treated cells.**


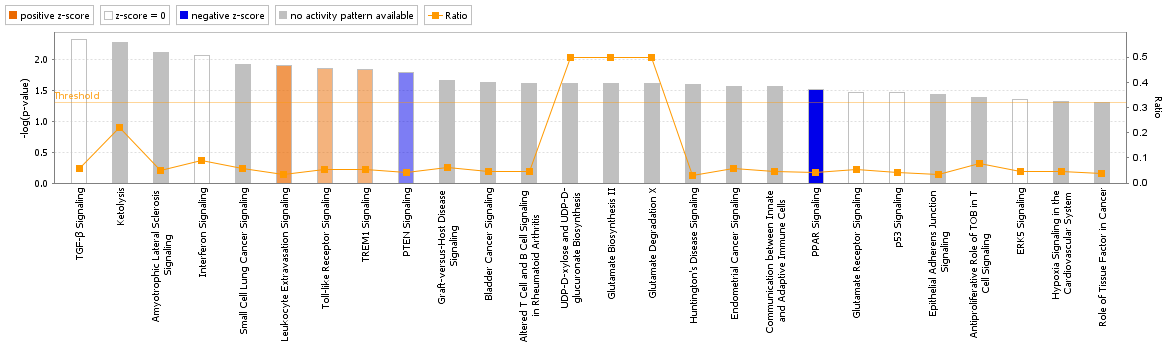


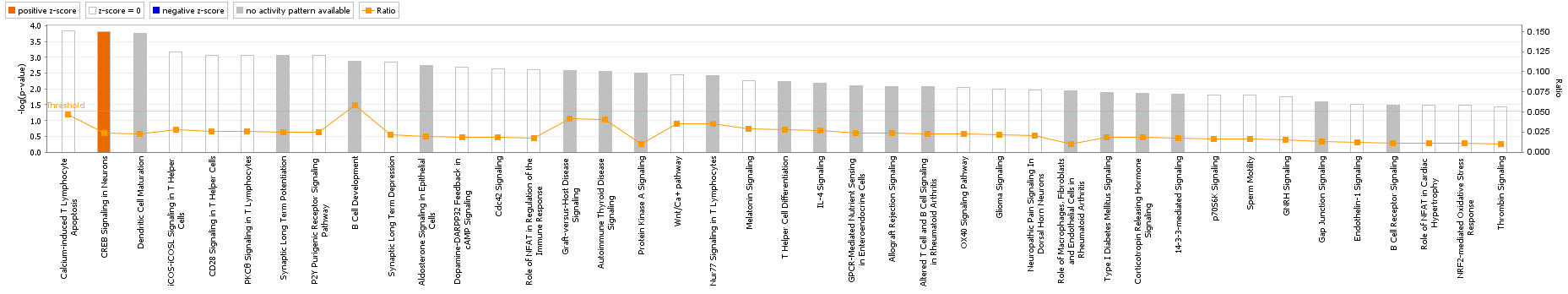

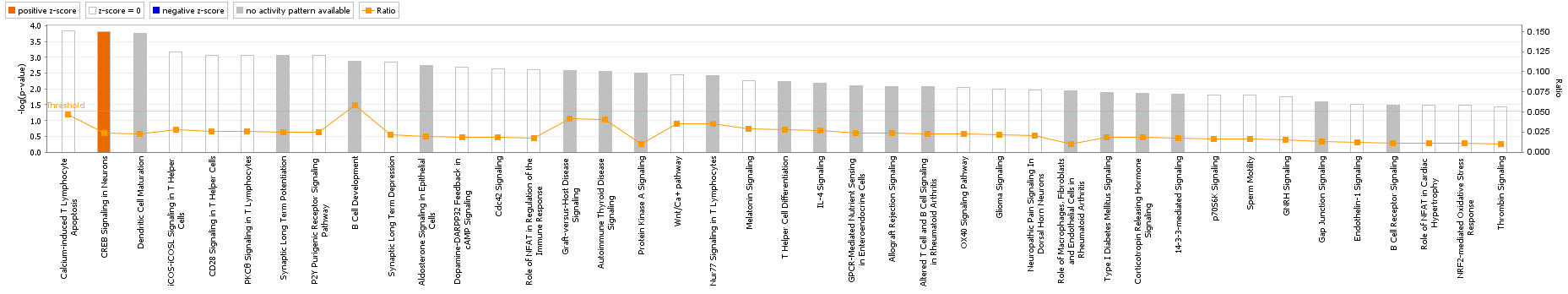


A

B
