# Supplementary material for: Differential Epigenetic Effects of Atmospheric Cold Plasma on MCF-7 and MDA-MB-231 Breast Cancer Cells
Source: PLoS One. 2015 Jun 4;10(6):e0129931. doi: 10.1371/journal.pone.0129931 (PMC4456358; doi:10.1371/journal.pone.0129931)
Supplement: S1 Table — (DOCX) [file pone.0129931.s005.docx]

| **S1 Table. Sequences of primers employed in this study.** | | |
| --- | --- | --- |
| **Genes** | **Forward primer (5’–3’)** | **Reverse primer (5’–3’)** |
| **Real-time RT-PCR** | |  |
| *PRR7* | CCAAGCGGAATCGGACAT | GGCTGCTCCTGCTTCTCC |
| *ESR1* | GTGCCAGGCTTTGTGGATT | GGTTCCTGTCCAAGAGCAAG |
| *CD86* | GCTTGCTAACTTCAGTCAACCTG | TCTGCATAACACCATCATACTCG |
| *FDX1* | AACAGACAGATCACGGTTGG | TTCCATGAAAATATTCCTATTTGTTCT |
| *CREB3* | TCTCACTAAGACAGAGGAACAAATTC | AGCTCCATATTCTGGGCTGT |
| *DHRS7B* | CATATGCAGCCTCCAAGCAC | CATAACTCCATACCTAGATCCATCC |
| *DNAJC8* | CGGCAGTTATCCATCTTGGT | CGCTCTTTCACAGTGTGTTCC |
| *POTED* | GATATGGAAGAACTGCCCTCA | AATTCACAAATTACATGATGATGAC |
| *EIF1AY* | GACAAGAGTATGCTCAGGTAATCAA | TTTGTTATCCTGATAGTCCCGTAGA |
| *BCL2* | GTGAACTGGGGGAGGATTGT | CATCCCAGCCTCCGTTATC |
| *BNDF* | CTACAGAAAGGGTTGCAGGTC | CTGGTGGAACTTTATGAAACCA |
